# Peptide-Based Targeting of the Platelet-Derived Growth Factor Receptor Beta

**DOI:** 10.1007/s11307-012-0578-7

**Published:** 2012-07-13

**Authors:** Vasileios Askoxylakis, Annabell Marr, Annette Altmann, Annette Markert, Walter Mier, Jürgen Debus, Peter E. Huber, Uwe Haberkorn

**Affiliations:** 1 Department of Radiation Oncology, University of Heidelberg and German Cancer Research Center, Im Neuenheimer Feld 400, 69120 Heidelberg, Germany; 2 Department of Nuclear Medicine, University of Heidelberg and German Cancer Research Center, Heidelberg, Germany

**Keywords:** Phage display, Peptide ligand, Targeting, Angiogenesis

## Abstract

**Purpose:**

The aim of this work is to identify new ligands targeting the platelet-derived growth factor receptor beta (PDGFRβ).

**Procedures:**

Biopanning was carried out with a 12-amino-acid phage display library against the recombinant extracellular domain of PDGFRβ. The identified peptide PDGFR-P1 was chemically synthesized and labeled with ^125^I or ^131^I. *In vitro* studies were performed on the PDGFRβ-expressing cell lines BxPC3 and MCF7 and on PDGFRβ-transfected HEK cells in comparison to negative control wtHEK293 and CaIX-transfected HEK cells. Biodistribution experiments were performed in Balb/c nude mice, carrying subcutaneously BxPC3 tumors.

**Results:**

*In vitro* studies demonstrated a higher binding to BxPC3, MCF7, and PDGFRβ-tr-HEK cells in comparison to negative control cell lines. Binding was inhibited up to 90% by the unlabeled PDGFR-P1 peptide. Organ distribution studies revealed a higher accumulation in BxPC3 tumors than in most organs.

**Conclusions:**

PDGFR-P1 is a promising candidate for targeting human PDGFRβ.

## Introduction

The platelet-derived growth factor receptor β (PDGFRβ) is a transmembrane glycoprotein that belongs to the receptor tyrosine kinase family [1]. Activation through the PDGF ligand leads to receptor dimerization, allowing the phosphorylation of regulatory tyrosine residues. Phosphorylated receptor tyrosine residues bind SH2 domain-containing proteins and initiate cytoplasmic signal transduction pathways that influence cell migration, proliferation, and differentiation [2, 3].

PDGFRβ is found to be upregulated in various solid tumors. The receptor is implicated in tumor growth mainly through angiogenesis activation. The role of PDGF-B/PDGFRβ signaling pathway in angiogenesis has been extensively investigated in knockout animal models [4]. Such studies revealed that PDGFRβ expressing pericytes are recruited by PDGF-B-producing endothelial cells to angiogenic vessels, leading to stimulation of vascular smooth muscle cells and therefore vessel maturation [5]. In addition, signaling of PDGFRβ has a key role in the regulation of tumor interstitial fluid pressure [6]. Preclinical data have revealed that targeting PDGFRβ results in a decrease of the interstitial fluid pressure, allowing a better transcapillary molecular transport [7]. This effect has been the basis for the initiation of clinical investigations of therapeutic approaches combining chemotherapy drugs with PDGFR inhibitors [8].

The PDGF/PDGFR pathway has also a pivotal role in radiation treatment outcome and toxicity. It has been shown that PDGFR inhibitors enhance the antiangiogenic effects of irradiation [9, 10]. Furthermore, PDGF signaling is involved in the pathogenesis of radiation-induced pulmonary fibrosis, with studies showing that treatment with receptor tyrosine kinase inhibitors could attenuate the development of therapy-related lung fibrosis [11]. Additionally, clinical investigations revealed that PDGFRβ is not only strongly expressed in different tumor types but that the protein expression is important for disease prognosis [12]. A recent analysis has demonstrated significant associations between the PDGFRβ status in prostate carcinomas with histopathological tumor characteristics, such as Gleason score and tumor stage and clinical characteristics, including survival [13].

The role of PDGFRβ in various pathophysiological mechanisms, including both tumor development and treatment outcome, makes the receptor an attractive structure for molecular targeting and imaging approaches. Ligands with receptor specificity might be used for visualization of angiogenesis, but also for imaging and prediction of treatment response. Within the past years, a number of PDGFRβ inhibitors targeting the kinase activity have been developed and tested both in preclinical and clinical studies, showing promising antitumor activity [14, 15]. However, most of those inhibitors are not specific for PDGFRβ, but also show activity to other kinases such as PDGFRα, BCR-ABL, c-kit, or VEGF receptor.

Antibodies represent ligands with high target specificity. Monoclonal antibodies (mAbs) or single chain variable antibody fragments (scFv) targeting PDGFRβ have been generated and investigated, demonstrating high target affinity and specificity [16, 17]. However, antibodies are known to possess pharmacokinetic properties that might be disadvantageous for targeting or imaging applications. Due to their high molecular weight, antibodies show a slow extravasation from the blood supply and a limited, inhomogenous tumor penetration [18]. Furthermore, despite the development of chimeric or humanized antibodies, the *in vivo* use of antibodies is often limited by their immunogenic potential [19]. As tools for imaging antibodies show unfavorable characteristics, they are slowly cleared from the blood, resulting in high background activity for extended time periods and reduced imaging contrast due to low signal-to-noise ratios [20].

Peptides are an attractive alternative to antibodies because of their advantageous pharmacokinetic properties for tumor targeting and imaging such as homogenous tumor penetration, reduced immunogenic potential, and rapid blood clearance, whereas they are easier and less expensive to generate [21]. Identification of peptides with specific targeting characteristics that could be used for both therapeutic and diagnostic approaches is subject of intensive investigation in ligand-related cancer research.

The aim of the present study is to identify peptides with affinity and specificity for the extracellular domain of the human platelet-derived growth factor receptor β. Selection panning was performed independently on immobilized target protein and protein in suspension using the phage display technology. The identified peptide ligand for both independent biopanning processes was chemically synthesized, radiolabeled, and characterized *in vitro* for affinity and specificity. *In vivo* organ distribution studies were performed in mice bearing PDGFRβ overexpressing tumors, and the peptide stability in human serum was investigated.

## Results

### Selection of Peptides Binding the Extracellular Domain of PDGFRβ

**Fig. 1:**
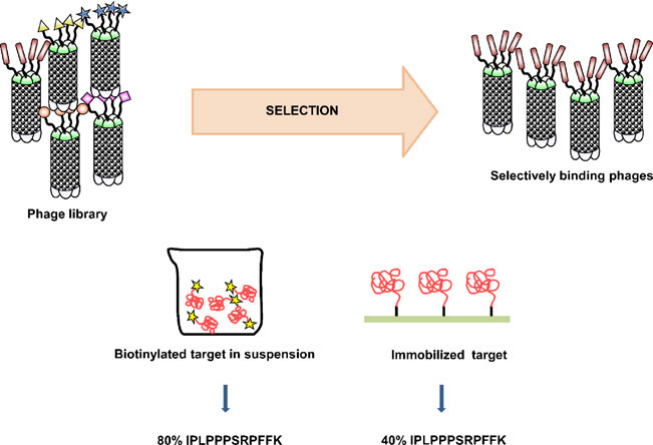
Schematic representation of the panning strategies applied on the extracellular domain of PDGFRβ. After four selection rounds using different negative control targets (EGFR and FGFR), the dodecapeptide IPLPPPSRPFFK was identified by both strategies.

To identify human PDGFRβ specific binding peptides, phage display was applied on immobilized recombinant extracellular domain of PDGFRβ and on biotinylated target in suspension. Extracellular domains of EGFR and FGFR were used as negative control targets. After four selection rounds, the same sequence was identified in both panning strategies. In particular, 40% of the clones isolated on immobilized protein and 80% of the clones isolated on biotinylated target in suspension displayed the peptide sequence: IPLPPPSRPFFK (Fig. 1).

### Binding Experiments on Immobilized Protein

**Fig. 2:**
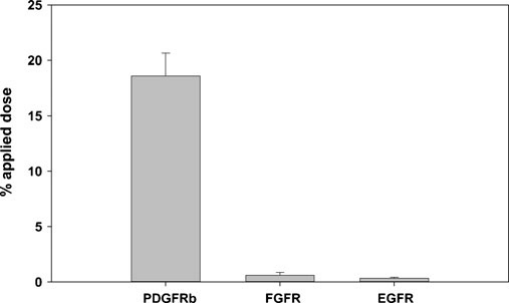
Binding of ^125^I-labeled PDGFR-P1 on the recombinant extracellular domain of PDGFRβ, EGFR and FGFR. Mean values and standard deviation.

The identified peptide including a C-terminal tyrosine residue for radiolabeling (PDGFR-P1 = IPLPPPSRPFFKY-NH_2_) was chemically synthesized, labeled with ^125^I and investigated on immobilized extracellular domains of PDGFRβ, EGFR and FGFR. Binding of the radioligand was about 18% on the extracellular domain of PDGFRβ. Experiments on the negative control targets (EGFR and FGFR) revealed only binding at the background level (*p* < 0.05) (Fig. 2).

### *In Vitro* Binding Experiments on PDGFRβ-Transfected Cells

**Fig. 3:**
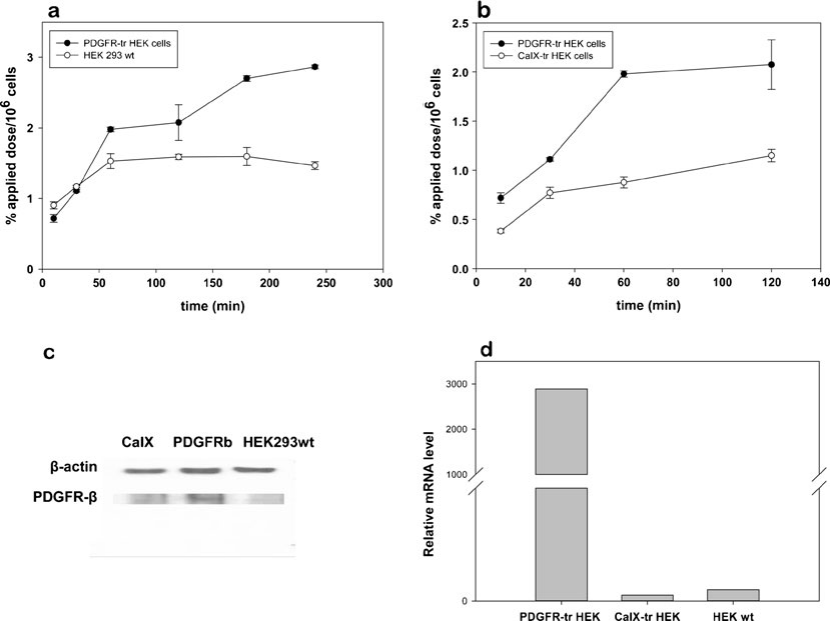
*In vitro* kinetics of ^125^I-labeled PDGFR-P1 in recombinant HEK293 cells stably overexpressing PDGFRβ (PDGFR-tr-HEK) in comparison to wild-type HEK293 cells (**a**) and negative control HEK cells transfected with human carbonic anhydrase IX (CaIX-tr-HEK) (**b**). Mean values and standard deviation. Expression of PDGFRβ in PDGFR-tr-HEK, HEK293wt, and CaIX-tr-HEK cells as investigated by Western blot (**c**) and real-time PCR analysis (**d**)

*In vitro* binding of ^125^I-labeled PDGFR-P1 was investigated on transfected HEK293 cells stably expressing PDGFRβ (PDGFR-tr-HEK). Wild-type HEK cells (HEK293wt) and human carbonic anhydrase IX (CaIX) transfected cells (CaIX-tr-HEK) were used as negative control targets. Kinetic studies revealed a significantly increased binding of PDGFR-P1 on PDGFR-tr-HEK cells compared to the negative controls (Fig. 3a, b) (*p* < 0.05). Peptide binding correlated with the expression of PDGFRβ in the three cell lines as evaluated by Western blot analysis (Fig. 3c) and real-time PCR (Fig. 3d).

### *In Vitro* Binding, Kinetic and Competition Experiments on BxPC3 and MCF7 Cells

**Fig. 4:**
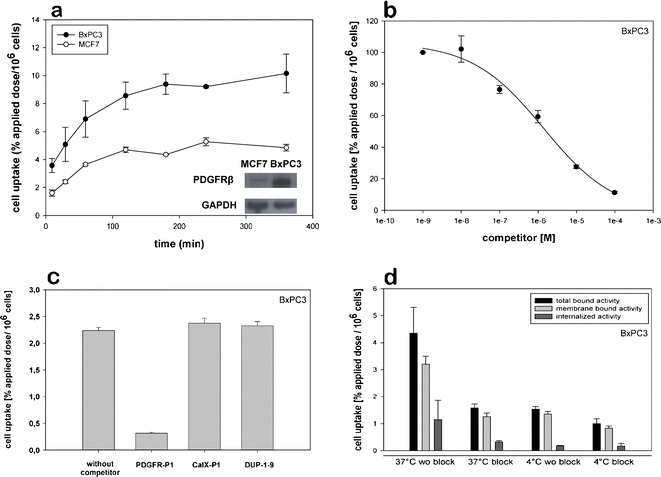
*In vitro* characterization of ^125^I-labeled PDGFR-P1. **a** *In vitro* kinetics on human pancreatic cancer BxPC3 and human breast cancer MCF7 cells. Peptide binding correlated with the target expression as characterized by Western blot analysis. **b** Displacement of bound ^125^I-labeled PDGFR-P1 by unlabeled PDGFR-P1 at various concentrations on BxPC3 cells. **c** Specific binding of ^125^I-labeled PDGFR-P1 on BxPC3 cells. Non-specific binding was determined in the presence of 10^−5^ M unlabeled PDGFR-P1. The peptides CaIX-P1 and DUP-1–9 were randomly used as negative control competitors at the same concentration. **d** Binding and internalization of ^125^I-labeled PDGFR-P1 in BxPC3 cells. Cells were incubated with the radioligand for 60 min at 37°C or at 4°C. The unlabeled peptide was used as competitor at a concentration of 10^−5^ M. Mean values and standard deviation (*n* = 3).

*In vitro* binding of ^125^I-labeled PDGFR-P1 was investigated on the human pancreatic carcinoma cell line BxPC3 and the human breast cancer cell line MCF7. After cell blocking with milk powder, cells were incubated with the radioligand in serum-free medium to avoid peptide degradation. *In vitro* kinetic experiments demonstrated an increase of radioligand binding with time. In particular, a maximal binding capacity of 9.5% and 5.2% applied dose per 10^6^ cells was measured for BxPC3 and MCF7 cells, respectively, which remained stable for the investigated period of 6 h (Fig. 4a). Peptide binding correlated with the expression of PDGFRβ in BxPC3 and MCF7 cells as evaluated by Western blot analysis (Fig. 4a). Co-incubation of ^125^I-labeled PDGFR-P1 on BxPC3 cells with the unlabeled peptide at various concentrations resulted in a concentration-dependent inhibition of radioligand binding, which reached a level of 90% at a competitor concentration of 10^−4^ M (*p* < 0.01). The IC_50_ value was calculated to be 1.4 μM (Fig. 4b). Competition experiments were performed using random peptides as negative control competitors, demonstrating that radioligand binding could be strongly inhibited by unlabeled PDGFR-P1 but not by the other competitors, such as CaIX-P1 and DUP-1-9 (Fig. 4c).

### Internalization Experiments

*In vitro* internalization was investigated in BxPC3 cells. After 60-min incubation with ^125^I-labeled PDGFR-P1, the internalized radioactivity was measured to be 26% of the total bound activity. Co-incubation with the unlabeled peptide led to a significant reduction of both total and internalized radioactivity (*p* < 0.05). Incubation at 4°C resulted in a 65% reduction of the total bound activity and a reduction of about 85% of the internalized activity. The measured internalized activity was 1.15% applied dose per 10^6^ cells at 37°C and 0.18% at 4°C (Fig. 4d).

### Stability in Human Serum

**Fig. 5:**
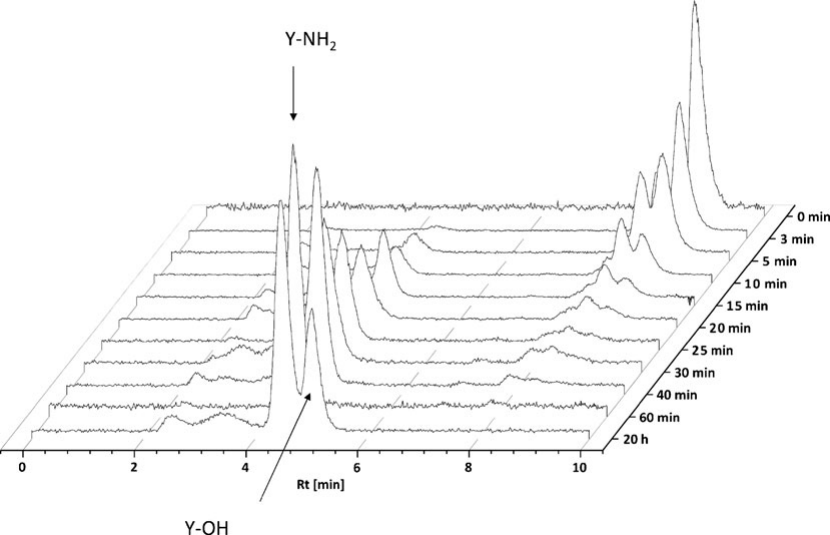
Serum stability analysis of ^125^I-labeled PDGFR-P1. HPLC analysis of aliquots collected at time points from 0 to 20 h.

Stability experiments revealed a rapid peptide degradation through serum proteases. The serum half-life of ^125^I-labeled PDGFR-P1 was 4 min. Degradation product analysis revealed a cleavage of the C-terminal tyrosine over time (Fig. 5).

### Organ Distribution Studies

**Fig. 6:**
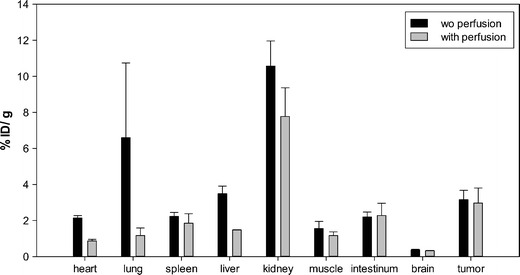
Organ distribution of ^131^I-labeled PDGFR-P1 in Balb/c nu/nu mice carrying BxPC3 tumors. *Black columns*—activity concentration (% ID/g) in tumor and control organs after 15-min circulation. *Gray columns*—radioactivity concentration (% ID/g) in tumor and control organs after perfusion of the animals (*n* = 3 animals per experiment).

Biodistribution experiments of ^131^I-labeled PDGFR-P1 were performed in mice, carrying subcutaneously transplanted BxPC3 tumors. The organ distribution revealed a tumor accumulation of 3.2% ID/g tissue after 15-min circulation. Tumor binding was higher compared to most healthy tissues (Fig. 6). The values in blood and kidney were higher (5.4% and 10.6% ID/g, respectively). To reduce blood background in both tumor and healthy tissues, biodistribution experiments were followed by perfusion with 0.9% NaCl. The perfusion experiments revealed a reduction in most healthy tissues, such as heart, lung, spleen, liver, kidney, and muscle but not in the tumor. This resulted in an increase of the tumor-to-organ ratios (Table 1), which was found to be highly significant for heart, lung, liver, kidney, and muscle (*p* < 0.01). In particular, the tumor-to-liver ratio was 0.906 before perfusion and 2.003 after perfusion (*p* < 0.05). The tumor-to-intestinum ratio showed no significant increase after animal perfusion.

**Table 1:** Tumor-to-organ ratios calculated from the organ distribution of ^131^I-labeled PDGFR-P1 in Balb/c nu/nu mice carrying BxPC3 tumors before and after organ perfusion (*n* = 3 animals per experiment)

Tumor-to-organ ratio	Without perfusion	After perfusion
Heart	1.474	3.373
Lung	0.567	2.882
Spleen	1.415	1.601
Liver	0.906	2.003
Kidney	0.299	0.379
Muscle	2.154	2.530
Intestinum	1.431	1.321
Brain	8.702	9.083

## Discussion

The platelet-derived growth factor receptor beta is a transmembrane glycoprotein belonging to the tyrosine kinase family. Protein stimulation by PDGF-B induces the recruitment of pericytes and their integration in the vascular wall, promoting angiogenesis [22]. PDGFRβ is expressed in various solid tumors and is involved in important biological processes, such as cell proliferation and regulation of the tumor interstitial pressure [23].

The fact that PDGFRβ is expressed in malignant tumors and that it is a transmembrane molecule and therefore easily accessible makes the protein an attractive target for the development of molecular targeting strategies for diagnosis and therapy. In regard to molecular imaging, non-invasive PDGFRβ visualization in solid tumors is important since it could optimize treatment monitoring, allowing a detailed evaluation of tumor response to therapeutic modalities.

A promising screening technology for identification of specific binding ligands is the phage display technology. Recently, affibody molecules, consisting of 58-amino-acid three helical polypeptides with affinity for PDGFRβ, were isolated using phage display selection [24]. The identified binders were highly specific, revealing reactivity only for human and murine PDGFRβ and demonstrating a potential for protein targeting.

However, although affibody molecules are very attractive alternatives to mAbs, there is still a need for development of even smaller peptides for use in targeted approaches. Oligopeptides are characterized by advantageous characteristics attributed to their small size, such as rapid blood clearance and increased tumor penetration. Therefore, the aim of the present study was to identify small peptide ligands with specificity and affinity for PDGFRβ. To isolate ligands with high target specificity, two different phage display strategies were performed independently from each other. Within the first strategy, panning was carried out on the immobilized extracellular domain of PDGFRβ, whereas the extracellular domain of EGFR was used as negative control target. Within the second strategy, panning was performed on the extracellular domain of PDGFRβ in suspension and a different negative control target (FGFR) was used. After several selection rounds, the same dodecapeptide (IPLPPPSRPFFK) was identified for both strategies, indicating a high PDGFRβ affinity.

Specific ligand binding on PDGFRβ was demonstrated in binding experiments using the radiolabeled, chemically synthesized PDGFR-P1 peptide. In particular, experiments on immobilized proteins showed a significantly increased binding on the extracellular domain of PDGFRβ compared to the extracellular domains of the negative control targets EGFR and FGFR. Furthermore, a significantly increased ligand binding was demonstrated on transfected HEK293 cells, stably overexpressing PDGFRβ, compared to both wild-type HEK293 but also to negative control HEK293 cells transfected with human carbonic anhydrase IX. Binding on transfected and wild-type cells was found to correlate with the expression of PDGFRβ as investigated both with real-time PCR and Western blot analysis, which further strengthens the hypothesis of specific target binding.

To determine the properties of PDGFR-P1 on tumor cells, the peptide binding was evaluated on human pancreatic cancer BxPC3 and human breast cancer MCF7 cells. BxPC3 cells were chosen because this cell line is well established and known to overexpress PDGFRβ [25]. The results of our studies on BxPC3 and MCF7 cells were in concert with the previous results and supported the hypothesis of specific target binding. Kinetic analysis on both cell lines revealed an increased binding on the strong PDGFRβ expressing BxPC3 cells and a correlation to the protein expression as investigated by Western blot analysis. Competition experiments on BxPC3 cells using the unlabeled peptide at various concentrations revealed a concentration dependent inhibition of radioligand binding. Furthermore, radioligand binding could be inhibited by the unlabeled PDGFR-P1 peptide, but not by other, randomly chosen, phage display peptides, such as CaIX-P1 [26] or DUP-1–9 [27].

Although the *in vitro* results indicate a PDGFRβ specificity, organ distribution analysis is necessary prior to the use of a ligand for *in vivo* applications. In this respect, a higher accumulation in the tumor, compared to the healthy tissues and blood, is a prerequisite. Our *in vivo* biodistribution studies showed a higher accumulation in the tumor than in most healthy tissues. In addition, perfusion experiments resulted in a significant accumulation decrease in most healthy tissues but not in the tumor, leading to a significant increase of the tumor-to-organ ratios. The intestinal accumulation did not decrease after animal perfusion, which might be explained by the expression of PDGF receptors in intestinal myofibroblasts [28]. The fact that the radioactivity level in the tumor remained constant after perfusion, whereas it strongly decreased mainly in highly perfused organs, such as heart, lung, and liver is evidence for a specific tumor binding, but only an unspecific accumulation in healthy organs as a result of a high blood pool contribution. The high blood background represents a major drawback in the use of the ligand for imaging purposes since it results in enhanced background noise. A possible explanation for the high blood levels is an interaction of the ligand with serum proteins, such as albumin. A further explanation is the peptide’s serum instability. Metabolic studies revealed a rapid peptide degradation by serum proteases, resulting in circulation of radiolabeled fragments. Therefore, a major issue of further investigation is the metabolic stabilization of PDGFR-P1. A systematic approach to achieve this goal includes the identification of the degradation site in the peptide sequence and the application of targeted modifications that cannot be recognized by serum proteases, such as exchange of amino acids by unnatural amino acids (D- or N-methylated amino acids) [29], peptide acetylation or pegylation [30, 31], or grafting of the binding motif into a stable scaffold structure [32]. In addition, the IC_50_ value of 1.4 μM shows that an affinity improvement is needed. In this direction, peptide dimerization or multimerization can be applied. Peptide homodimers or homomultimers are usually characterized by improved target affinity compared to their monomers, mainly due to optimized cooperative receptor–ligand interactions as a result of increased ligand concentration [33]. Considering the dimeric nature of PDGFRβ, dimerization might also enhance the affinity of PDGFR-P1, a hypothesis that needs to be further investigated.

In conclusion, the results of our studies indicate that PDGFR-P1 might be a promising candidate for the development of novel peptide-based ligands, targeting the platelet-derived growth factor receptor beta. Such ligands could be used for visualization of PDGFRβ expression in tumors and their vasculature, as well as for monitoring treatment response. However, prior to the use of the peptide for imaging or targeting applications, it is of highest priority to improve its metabolic stability and promote the development of derivatives with optimized *in vivo* binding characteristics.

## Materials and Methods

### Cell Lines

All cell lines were cultivated at 37°C in a 5% CO_2_ incubator. The human pancreatic carcinoma cell line BxPC3, the recombinant cell lines PDGFRβ-tr-HEK and CaIX-tr-HEK, and wild-type HEK293 cells (HEK293wt) were cultivated in RPMI medium, supplemented with 10% FCS (Invitrogen). The human breast cancer cell line MCF7 was cultured in DMEM with Glutamax, containing 10% FCS (Invitrogen).

### Recombinant Isolation of the Extracellular Domain of Human PDGFRβ

The extracellular domain of PDGFRβ was recombinantly synthesized using the Flp-In system (Invitrogen Life Technologies) as described [26]. PCR amplification of the sequence encoding for the target was performed using a pCMV-SPORT6 vector including the PDGFRβ gene (ImaGenes, Germany). The primers for PCR amplification were forward: 5′-AAC TTA AGC TTG GGG CCG CCA CCA TGC GGC TTC CGG GTG CGA-3′ and reverse: 5′-GGC TCC GGA TCC ATG TCC CTG CCC TCG ATC TTA AAG GGC AAG GAG TGT GG-3′. After PCR amplification, the sequence was inserted into the HindIII and BspEI sites of a pSEC-EGP-2-Fcγ vector (Affimed, Heidelberg, Germany). The fragment PDGFRβ-Fc was cut by HindIII and XhoI, inserted into a pcDNAEpcam vector (Affimed), and transfected with the Flp recombinase expression vector pOG44 into the Flp-In^TM^-293 human embryonic kidney host cell line. After selection for hygromycin resistance, cells excreting the fusion protein were seeded in CELLine AD 1000 flasks (Integra) and 5 L of culture medium were collected. Protein purification was carried out using a HiTrap^TM^ MabSelect SuRe^TM^ column, and Western blot analysis as well as ELISA was applied for qualitative control (data not shown).

### Establishment of a Cell Line Permanently Expressing PDGFRβ

PCR amplification of the coding sequence of PDGFRβ was carried out. The primers used were forward: 5′-GAT GAT ATC ATG CGG CTT CCG GGT GCG A-3′ and reverse: 5′-ATC GAT ATC CTA CAG GAA GCT ATC CTC TGC-3′. The amplified sequence was digested with EcoRV and inserted into the SmaI site of pSKEF1αIIIVSIREShyg vector (own lab construct). The construct was then cut by SalI blunt and NsiI. After insertion into the XhoI blunt and NsiI sites of M48EF1αDmdNkIVSIREShyg vector (own lab construct), stable transfection of HEK293 cells was performed, using the LipofectAMINE PLUS Reagent package (Invitrogen Life Science), according to the manufacturer’s protocol. RNA was isolated using the TRIzol method (TRIzol Reagent, Invitrogen) and mRNA expression of PDGFRβ was assessed by real-time PCR as described below. Protein expression from selected clones was verified by Western blot analysis as described below.

### Selection of Peptides Binding PDGFRβ

Peptide selection was performed using a commercial 12-amino-acid peptide library (Ph.D.12; New England Biolabs). The recombinant extracellular domain of PDGFRβ was used as target, either immobilized in 96-well plates or biotinylated in suspension. Immobilized recombinant extracellular domain of the epidermal growth factor receptor (EGFR) and biotinylated extracellular domain of the fibroblast growth factor receptor (FGFR) were used for negative selection, respectively.

### Panning on Immobilized Target

Each selection round was conducted as previously described [26]: 10^11^ plaque-forming units were added on immobilized negative target (EGFR) in 96-well plates. After incubation for 1 h at room temperature, medium containing unbound phages was transferred on the immobilized positive target (PDGFRβ) and further incubated for 1 h at room temperature. Thereafter, medium was removed, the positive target was washed 10 times with 100 μl TBST and the bound phages were eluted through incubation with 10 μl 0.2 M glycine/HCl buffer pH 2.2, containing 1 mg/ml BSA. The eluent was neutralized with 15 μl Tris/HCl buffer pH 9.1, and centrifuged for 5 min at 1,000 rpm. Ten microliter supernatant aliquots were used for phage titration on IPTG/X-Gal (Fermentas) lysogeny broth agar plates.

### Panning with Biotinylated Target in Suspension

Both positive (PDGFRβ) and negative (FGFR) target were biotinylated as follows: 20 μl of 10 mM EZ-LinkSulfo-NHS-LC-Biotin (Pierce, Rockford, IL, USA) was added to 1 ml target in PBS pH 7.4 and mixed well. The biotinylation was accomplished when the mixture stood at 4°C for about 2 h. The free biotin reagent was removed by dialysis in PBS pH 7.4 at 4°C. Each selection round was conducted as follows: 10^11^ plaque-forming units were added in 500 μl PBST supplemented with 0.1% BSA pH 7.0. After 30-min incubation, 50 μl Dynabeads® M-280 Streptavidin (Invitrogen) was added together with 100 μl biotinylated negative target FGFR 100 nM and 100 μl non-biotinylated FGFR 1,000 nM. After 1-h incubation at room temperature, the unbound phages were separated with a Dynal magnet and incubated with 100 μl biotinylated PDGFRβ for 1 h at room temperature. Fifty microliters of Dynabeads® M-280 Streptavidin was added and after further 15-min incubation the PDGFRβ-bound phages were separated with a Dynal magnet. The beads were washed with 1,000 μl PBST/0.1% BSA pH 7.0 and the bound phages were eluted with 1000 μl 0.2 M glycine/HCl buffer pH 2.2. The magnetic beads were separated with the Dynal magnet and neutralization was performed with 15 μl Tris/HCl buffer pH 9.1. Supernatant was collected and 10 μl was used for phage titration on IPTG/X-Gal (Fermentas) lysogeny broth agar plates.

### Phage Amplification and Sequencing

Bound phages were amplified in 20 ml of ER2537 bacteria according to the manufacturer’s protocol. After four selection rounds, single-stranded phage DNA was isolated from picked clones using a QIAprep Spin M13 Kit (Qiagen). The sequence of the displayed peptide was identified through DNA sequencing and analysis using the HUSAR map (HUSAR Biocomputing Service at the German Cancer Research Center), as previously described [26].

### Peptides

The peptide PDGFR-P1 (IPLPPPSRPFFKY-NH_2_) was synthesized on an ABI433A peptide synthesis reactor (Applied Biosystems) using Fmoc coupling protocols. Peptide purification was performed by high performance liquid chromatography (HPLC) on a Chromolith Semi Prep Column RPe18, 10 × 100 mm (Merck), with a linear gradient of water and acetonitrile containing 0.1% trifluoroacetic acid. After lyophilization of the product, mass analysis was performed on a matrix-assisted laser desorption ionization time-of-flight mass spectrometer (MALDI-3; Kratos instruments). The chloramine-T method was used for peptide labeling with ^125^I or ^131^I [34]. HPLC purification of the iodinated product was carried out on a Chromolith Performance RP-18e 100 × 4.6 mm column (Merck). The specific activities for the ^125^I- and the ^131^I-labeled peptides were about 50 GBq/μmol.

### Binding Experiments on Immobilized Protein

Binding of ^125^I-labeled PDGFR-P1 was investigated on immobilized extracellular domain of human PDGFRβ, EGFR, and FGFR. The target proteins were incubated at a concentration of 25 nM in 96-well Maxisorp^TM^ plates (Nunc GmbH & Co. KG, Germany) for 24 h and the plates were washed thrice with 100 μl PBS pH 7.4. ^125^I-labeled PDGFR-P1 was incubated with the target in 100 μl PBS pH 7.4 for 60 min. The unbound activity was removed by washing with 300 μl ice-cold PBS pH 7.4. Bound radioligand was determined through degradation with 100 μl NaOH 0.3 M and radioactivity measurement. Radioligand binding was calculated as percentage applied dose.

### *In Vitro* Binding Experiments

Three hundred thousand BxPC3, MCF7, PDGFRβ-tr-HEK, CaIX-tr-HEK, or HEK293wt cells were cultivated in six-well plates at 37°C for 24 h. Cells were blocked with RPMI 1640 containing 1% milk powder and the medium was replaced with 1 ml fresh medium (without FCS) containing 0.5–1.5 × 10^6^ cpm ^125^I-labeled peptide. After incubation at 37°C for various time periods (10 min to 6 h), the medium was removed and the cells were washed with PBS. Subsequently, cell lysis was performed with 0.5 ml NaOH 0.3 M and the bound radioactivity was measured with a γ-counter and calculated as percentage applied dose per 10^6^ cells. Specific binding was determined through co-incubation of the radioligand with the unlabeled PDGFR-P1 peptide at various concentrations (10^−4^ to 10^−10^ M). A dodecapeptide identified with phage display by panning against the extracellular domain of human carbonic anhydrase IX (CaIX-P1) [26] as well as a derivative of a dodecapeptide identified with phage display on prostate carcinoma cells (DUP-1–9) [27] were used as negative control competitors.

### Internalization Studies

Subconfluent cell cultures of BxPC3 cells were incubated with ^125^I-labeled PDGFR-P1 for 60 min at 37°C and 4°C. After cell washing with PBS, incubation was performed with 1 ml glycine–HCl 50 mmol/l in PBS (pH 2.8) for 10 min at room temperature in order to remove the membrane bound activity. Internalized activity was determined through subsequent cell lysis with 0.5 ml NaOH 0.3 M and measurement with a γ-counter as previously described.

### Stability Studies

The stability of PDGFR-P1 was investigated in human serum. ^125^I-labeled peptide was incubated at 37°C in human serum. At time points varying from 5 min to 20 h, aliquots were taken, mixed with equal volume acetonitrile, to precipitate serum proteins and centrifuged for 5 min at 13,000 rpm. Serum degradation products were determined by HPLC analysis of the supernatant.

### *In Vivo* Experiments

Organ distribution studies were carried out in 9-week-old female Balb/c nu/nu mice (Charles River WIGA), carrying subcutaneously transplanted human pancreatic cancer BxPC3 tumors. Tumor implantation was performed through injection of a cell suspension (4 × 10^6^ cells in OPTI-MEM) (Gibco, Invitrogen Life Technologies) into the mouse trunk. After the tumors were grown to a size of approximately 1.0 cm^3^, 1 MBq of ^131^I-labeled PDGFR-P1 was injected into the tail vein, and at 15 min p.i. the animals were euthanized. Tumor, blood, and healthy tissues were removed, weighed, and measured with a γ-counter (LB 951G; Berthold Technologies). Organ uptake calculation was performed as percentage injected dose per gram tissue (% ID/g). To avoid bias due to blood background radioactivity, uptake in blood-free organs was determined with perfusion experiments [35]. Within these experiments, blood was removed through a catheter in the ascending aorta of the anesthesized animal and perfusion with approximately 25 ml 0.9% NaCl was performed via a cut in the animal liver. After perfusion, samples of tumor and organs were removed and measured as described. All animal experiments were carried out in conformity with the German and European laws for animal protection.

### Real-Time Quantitative PCR

Total cellular RNA was isolated from confluent PDGFRβ-tr-HEK, CaIX-tr-HEK, and HEK293wt cells using the Trizol method (TRIzol Reagent, Invitrogen) and a standard phenol–chloroform RNA extraction protocol. Five hundred nanograms of RNA was transcribed into DNA using M-MLV reverse transcriptase, 50 pmol random hexamer, and 100 pmol of oligo(dT) primers (Promega, Madison, WI, USA). Relative mRNA transcript levels for PDGFRβ were quantified applying the TaqMan methodology on a StepOnePlus^TM^ Real-Time PCR System (Applied Biosystems). Normalization was performed using GAPDH as housekeeping gene. All primers were obtained from Applied Biosystems (Foster City, CA, USA).

### Western Blot Analysis

BxPC3, MCF7, HEK293wt, PDGFRβ-tr-HEK, and CaIX-tr-HEK cells were grown to 80% confluency. After cell washing with PBS and lysis with 1 ml cell lysis buffer (1 M Tris, 5 M NaCl, 1 mM EDTA, 1 mM EGTA, pH 8.01), centrifugation at 13,000 rpm was performed for 10 min at 4°C. The supernatant was collected for Western blot analysis as described [9]. In particular, protein transfer from a polyacrylamide gel to a nitrocellulose membrane was performed using a Mini Trans-Blotter (100 V for 90 min). Subsequently, the membranes were blocked with 5% non-fat milk powder in TBST buffer for 1 h at room temperature and incubation with a mouse IgG_1_ monoclonal anti-human PDGFRβ antibody (R&D Systems) was carried out overnight at 4°C. The nitrocellulose membrane was washed with TBST and incubated with a horseradish peroxidase conjugated antibody in blocking buffer at room temperature for 60 min. Antibody binding was determined using a chemiluminescence detection system with exposures recorded on hyperfilms for 10 s to 3 min.

### Statistics

Data were analyzed employing the paired two-tailed Student *t* test and significance was assumed at *p* <0.05.
